# Dr. James C. Knapp

**Published:** 1910-01

**Authors:** 


### OBITUARY

Dr. James C. Knapp, of Geneva, N. Y., a homeopathic physician of prominence in that region, recently died at his home in that city.

